# Vertical-flow porous microchamber arrays for cell capture, intracellular molecule staining, and population analysis

**DOI:** 10.1039/d5ra10009g

**Published:** 2026-04-22

**Authors:** Asahi Ohtsu, Yusuke Araki, Rie Utoh, Masumi Yamada

**Affiliations:** a Department of Applied Chemistry and Biotechnology, Graduate School of Engineering, Chiba University 1-33 Yayoi-cho, Inage-ku Chiba 263-8522 Japan m-yamada@faculty.chiba-u.jp +81-43-290-3398

## Abstract

Staining and visualization of intracellular molecules are fundamental processes for cell characterization, identification, and detection in medical diagnostics and general biological research. Conventional protocols for staining intracellular molecules require labor-intensive, multistep solution exchange procedures mostly based on centrifugation, which lead to significant cell loss and degrade the reproducibility and reliability of the results. In this study, we propose a microchamber array platform based on a porous substrate that enables intracellular molecule staining with minimal cell loss and low reagent consumption, while preserving cell morphology. Devices with porous microchambers were fabricated by combining PDMS-based replica molding and salt-leaching techniques. By a simple drop-based operation, vertical flow is passively generated through the porous substrate, enabling highly controlled, multistep reagent exchange for processing the captured cells. We performed dual staining of F-actin and cell nuclei for two types of mammalian cells with minimal reagent consumption. In addition, circulating tumor cell models were spiked into leukocyte samples and the detection, identification, and quantitative evaluation of rare cell populations were demonstrated. The proposed microchamber-based platform provides a generalizable and scalable solution for loss-free cell processing, with broad applicability in cell characterization, rare cell analysis, and diagnostic research.

## Abstract 2

Vertical-flow porous microchamber arrays enable low-loss cell capture, controlled multistep reagent exchange, and efficient intracellular staining for reliable population analysis with minimal reagent consumption.

## Graphical abstract

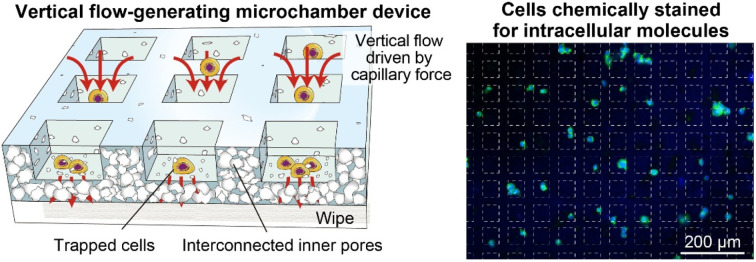

## 1. Introduction

The visualization of specific molecules inside and/or outside cells represents one of the most fundamental and universal approaches for analyzing target cell populations and their characteristics in complex biological samples. In particular, intracellular staining techniques that exploit specific biochemical interactions, such as antibody-mediated recognition, have played a central role in advancing both biological research and clinical diagnostics. For example, visualization of cytoskeleton structures such as actin and tubulin enables detailed analysis of dynamic cell morphology, allowing the evaluation of cell adhesion and the identification of cell types.^1–3^ Cytokeratin, a type of intermediate filament that constitutes the cytoskeleton of epithelial cells, plays a crucial role in epithelial–mesenchymal transition (EMT) of cancer cells.^4–6^ Analysis of the morphology and expression amount of these molecules is key to understanding metastatic state, chemoresistance, and recurrence in epithelial cancers.^7–9^ Beyond cytoskeletal analysis, staining and quantification of intracellular molecules are indispensable for elucidating intracellular signaling pathways,^10,11^ quantitatively evaluating cytokine production,^12^ and detecting apoptosis.^13^

To visualize target intracellular molecules, staining reagents must be delivered to the interior of cells across the cell membrane. This process requires a series of chemical treatments, including cell fixation, membrane permeabilization, and subsequent staining. Such multistep processing is indispensable, particularly for suspension cells, when performing quantitative analyses using flow cytometry.^14,15^ Conventional intracellular staining protocols typically involve repeated solution exchange steps, most commonly performed by centrifugation. However, centrifugation-based solution exchange often requires 10–20 rounds of manual handling,^16^ making the overall procedure labor-intensive and highly operator-dependent. As a result, substantial cell loss and variability in staining performance are frequently unavoidable. In addition, microfluidics-based solution exchange techniques have been reported for application to multistep staining processes.^17,18^ However, these approaches often rely on complex fluidic systems and may require relatively large amounts of staining reagents, limiting their practical utility. Alternative strategies involve trapping cells in microfabricated chambers placed in microchannels^19^ or confining cells within thin channels with depths comparable to cell size.^20^ Although these methods reduce cell loss and reagent consumption, their operation still requires careful handling of microchannels, including precise reagent/air introduction, limiting their widespread application. Therefore, there remains a strong demand for robust platforms that enable reliable cell capture and efficient solution exchange through simple operations, without relying on complex fluidic control systems.

To overcome these challenges, we focused on sponge-like materials with interconnected micropores. In recent years, numerous studies have reported the development and application of porous polymeric substrates, as well as their integration with microfluidic devices and microfabricated structures.^21–24^ Such porous substrates are typically fabricated by pre-introducing sacrificial porogens into elastic base materials, such as silicone, followed by their subsequent removal.^25^ These porous polymeric materials have been applied as oil-absorbing substrates,^26–28^ substrates for wearable electronics,^29,30^ and cell culture scaffolds.^31–33^ In our research group, we previously combined molding techniques with the formation of porous polydimethylsiloxane (PDMS) substrates using NaCl particles as sacrificial porogens. Using this approach, we fabricated flow-through porous microchamber array structures for the perfusion culture of mammalian cells.^34^ In this system, the pore size at the substrate surface can be readily tuned, enabling the formation of pores smaller than typical mammalian cells (approximately 10 µm). We anticipated that such a structure would enable direct delivery of staining solutions through the substrate while gently capturing cells, thereby providing a new strategy for cell processing. This approach was expected to eliminate labor-intensive procedures, such as repeated centrifugation, while simultaneously minimizing reagent consumption.

**Fig. 1:**
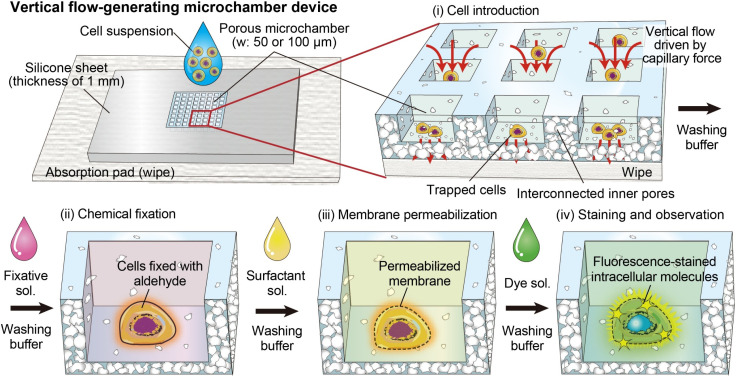
Schematic illustration showing the vertical flow-generating devices with porous microchambers for staining intracellular molecules of mammalian cells. Cells are first captured onto the chambers, then fixative solution, surfactant solution, and dye solution are sequentially applied.

The concept of the proposed device for cell capture and staining, together with its operation procedure, is illustrated in Fig. 1. This device consists of a planar silicone sheet in which the central area is composed of porous PDMS and formed with an array of microchambers. This porous region contains a three-dimensionally interconnected pore network. When a droplet of cell suspension is applied, the medium permeates through the porous substrate, while the cells are captured within the microchambers. By bringing the bottom surface of the device into contact with an absorbent wipe, the applied solution infiltrates into the porous substrate driven by capillary force. Consequently, solution exchange can be carried out through a simple drop-based operation, in which small volumes of processing reagents, including fixative solution, surfactant solution, and dye solution, are sequentially dispensed onto the device. The porous microchamber region has a square geometry with side lengths on the order of several millimeters. As a result, introduced cells are confined within a well-defined and localized area, which facilitates quantitative evaluation of cell populations, including determination of target cell ratios, from small-volume samples.

To evaluate the utility of the proposed system, we first fabricated porous microchamber arrays by integrating a salt-leaching method, in which NaCl particles were used as sacrificial porogens and subsequently removed from the substrate, with a conventional replica molding process.^34^ We then characterized the pore size and the fluid permeability of the porous microchambers. In addition, we examined capture behavior of fluorescent beads to examine whether the system could be applied to quantitative evaluation. Finally, we introduced cell suspensions and attempted cytoskeletal staining through the simple operation of sequentially dispensing the required reagents. Using a heterotypic cell sample, we further demonstrated quantitative evaluation of cancer cell populations based on intracellular molecule staining.

## 2. Experimental

### 2.1. Design and fabrication of microchamber devices

**Fig. 2:**
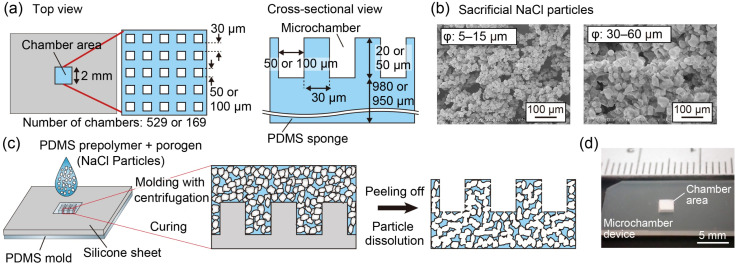
(a) Design of the devices and the chamber structures. (b) SEM images of the sacrificial NaCl particles used to form the porous substrate. (c) Schematic image showing the fabrication process of the devices. (d) Photograph of the fabricated device.

The design of the vertical flow-generating microchamber devices presented in this study is shown in Fig. 2(a). Two types of devices with different chamber sizes and numbers were designed and fabricated. These devices have a rectangular shape (10 × 20 mm) and a thickness of 1 mm. The central square area (2 mm × 2 mm) was composed of a porous PDMS substrate, within which an array of square-shaped chambers was formed. The chamber sizes were 50 µm and 100 µm, corresponding to a total of 529 (23 × 23) and 169 (13 × 13) chambers, respectively. The depth of the 50 µm-wide chambers was 20 µm, and that of the 100 µm-wide chambers was 50 µm, both corresponding to approximately half of the chamber width. The width of the walls separating the chambers was set to 30 µm for both devices.

These devices incorporating porous chambers were fabricated by applying a method that combines replica molding and salt-leaching.^34^ In this study, three types of NaCl particles with different size ranges (5–15 µm, 15–25 µm, and 30–60 µm; Naikai Salt, Okayama, Japan) were used as sacrificial porogens (Fig. 2(b)). The fabrication process is shown in Fig. 2(c). First, a PDMS mold with a convex chamber structure was fabricated by standard soft lithography using SU-8 and by repeating replica molding twice. A silicone sheet (10 mm × 20 mm, 1 mm thick) with a square hole (2 mm × 2 mm) cut at the center was then placed on the PDMS mold, aligning the convex chamber structures with the square hole. Next, the sacrificial NaCl particles, sieved using a stainless steel mesh (with a mesh size of 38 µm or 100 µm), were mixed thoroughly with PDMS prepolymer (Silpot 184, Dow Corning Toray, Tokyo, Japan, at a base and curing agent ratio of 10 : 1) at a volume ratio of 1 : 1, using a centrifugal mixer (AR-100, Thinky, Tokyo, Japan). The mixture was introduced into the hole of the silicone sheet and centrifuged vertically at 3500 rpm for 15 min using a plate centrifuge to eliminate air bubbles and ensure tight packing. After centrifugation, PDMS was cured by heating at 85 °C for 150 min in a convection oven. Following curing, the surface was flattened, and the structure was immersed in deionized (DI) water at 70 °C overnight to dissolve the NaCl particles. The device was then washed and dried.

### 2.2. Characterization of the microchamber devices

Several tests were performed to characterize the performance of the fabricated devices. First, the chamber morphology was examined using a scanning electron microscope (SEM; VE-8800, Keyence, Osaka, Japan). The pore sizes were quantitatively analyzed using image processing software based on the equivalent circular diameter corresponding to the same area. The permeability of the porous substrate was also evaluated by a water penetration test. As a pre-treatment, the device was immersed in DI water and degassed to completely remove air from the porous substrate and fill the pores with water. The device was then placed on a nonwoven wipe (Bemcot M-3II, Asahi Kasei, Tokyo, Japan), and 4 µL of DI water was dropped onto the porous area. The time required for the water to completely pass through the porous substrate was measured.

Furthermore, particle capture experiments were conducted. Fluorescent microparticles with a diameter of 9.9 µm (G-1000, Thermo Fisher Scientific, MA, USA), which served as model cells, were suspended in DI water at a concentration of 2.5 × 10^5^ particles per mL. Following the same procedure as in the water penetration test, 4 µL of the particle suspension was dropped onto the device. After the liquid completely passed through, 4 µL of distilled water was added to wash the chambers, and the washing step was repeated once more. The fluorescent particles trapped in the chambers were observed using an inverted fluorescence microscope (IX-71, Olympus, Tokyo, Japan), and the number of captured particles per chamber was counted.

### 2.3 Preparation of cells

Two types of mammalian cell lines were used. MCF-7 cells (a human breast cancer cell line, kindly provided by Riken BRC, Ibaraki, Japan) were cultured in DMEM supplemented with 10% (v/v) fetal bovine serum (FBS) and 1% (v/v) penicillin/streptomycin solution at 37 °C in a humidified atmosphere with 5% CO_2_. When the cells reached approximately 80% confluence, they were harvested *via* trypsin/EDTA treatment, collected by centrifugation, and resuspended in the culture medium. Jurkat cells (a human T lymphocyte leukemia cell line, obtained from ATCC, VA, USA) were cultured in RPMI-1640 medium supplemented with 10% (v/v) FBS and 1% (v/v) penicillin/streptomycin solution. For experiments, these cells were resuspended in the culture medium at a concentration of 1.25 × 10^5^ cells per mL.

### 2.4. Staining of F-actin

In the staining procedure for intracellular molecules, all liquid samples (cell suspension, cell processing/staining solutions) were applied dropwise in volumes of 4 µL for each step. In addition, the sample was washed two times with PBST between each step. When introducing these liquid samples into the microchamber device, a wipe was placed in contact with the bottom surface of the device. During incubation steps, the wipe was detached to temporarily stop reagent permeation, and the device was placed in a plastic Petri dish to prevent evaporation. All procedures were carried out at room temperature.

First, the device was immersed in saline and degassed to remove air from the porous substrate and fill the pores with saline. Then, 4 µL of a suspension of MCF-7 or Jurkat cells at a concentration of 1.25 × 10^5^ cells per mL (containing approximately 500 cells) was dropped onto the microchambers. Next, 4% paraformaldehyde (Fujifilm Wako Pure Chemical, Osaka, Japan) in PBS was dropped and incubated for 10 min to fix the cells. Subsequently, 0.5% Triton X-100 (Sigma-Aldrich, MO, USA) in PBS was applied and incubated for 5 min to permeabilize the cell membranes. Then, 4 µL of Acti-stain 488 fluorescent phalloidin in PBS (at a concentration of 100 nM, Cytoskeleton, CO, USA) was applied, and the device was incubated in the dark for 1 h to stain F-actin. Finally, 4 µL of Hoechst 33342 in PBS (10 µg mL^−1^, Thermo Fisher Scientific) was dropped and incubated for 2 min to stain the nuclei, followed by a washing step. After sealing the chambers with a cover glass, the stained cells were observed using the inverted fluorescence microscope. Circularity, projected cell area, and mean fluorescence intensity (MFI) of staining were quantified using ImageJ software and compared with those obtained using the conventional centrifugation-based solution exchange method.

### 2.5. Staining of CK-19 for identification of cancer cells

Trapping a heterotypic cell mixture and selective quantification of a rare cell population were performed. We prepared mixtures of MCF-7 cells and Jurkat cells with varying their ratios. The number of the Jurkat cells was kept constant (1000 cells per 4 µL of a suspension), while that of the MCF-7 cells was set to 0, 10, and 100 cells per 4 µL of the suspension. Cytokeratin-19 (CK-19), a molecule strongly expressed in most epithelial tumor cells,^35^ was stained for the MCF-7 cells, while the nuclei of these two cell types were stained blue. We followed the same procedure as described for F-actin staining until the cell membrane permeabilization step. Subsequently, 1% bovine serum albumin (Rockland Immunochemicals, PA, USA) in PBS was applied to the porous area and incubated at room temperature for 30 min for blocking. Next, rabbit anti-human CK-19 antibody in PBS (2 µg mL^−1^, Abcam, UK) was added and incubated for 1 h. Then, Alexa-Fluor 594 goat anti-rabbit IgG (4 µg mL^−1^, Thermo Fisher Scientific) was applied and incubated for 30 min in the dark to stain CK-19. Finally, cell nuclei were stained with Hoechst 33342, followed by washing, and the cells were observed under a fluorescence microscope.

## 3. Results and discussion

### 3.1. Fabrication of porous microchamber devices

For efficient cell trapping within microchambers and subsequent intracellular staining, the microchamber devices are required to satisfy several criteria. First, the pores formed at the bottom of the chambers must be sufficiently small to effectively capture cells. Second, the internal pores of the porous substrate should be interconnected throughout the thickness of the device to enable vertical transport of reagents. Third, pore formation on the upper surfaces of the walls separating adjacent chambers should be minimized to ensure that cells are selectively trapped within the chambers rather than on the walls. To examine whether the proposed fabrication method satisfied these requirements, we designed and fabricated two types of devices with different chamber sizes (50 or 100 µm square chambers) and evaluated the controllability of the number of cells trapped per chamber. In addition, to investigate the effect of sacrificial NaCl particle size on pore formation and fluid permeability, NaCl particles with three different size ranges were examined.

**Fig. 3:**
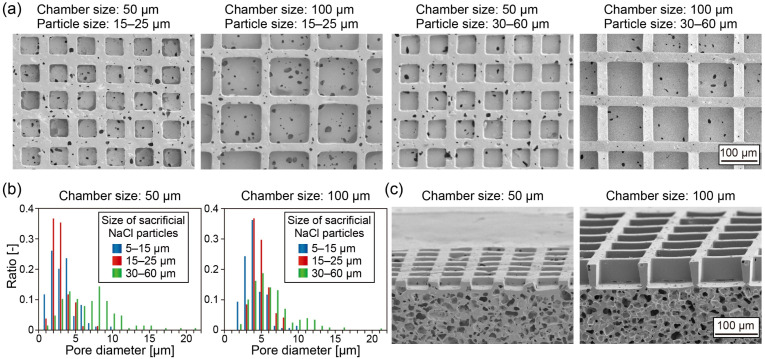
(a) SEM images of the chambers with different sized prepared using sacrificial NaCl particles with different sizes. (b) Distribution of pore diameter when the chamber size and the sacrificial NaCl particles sizes were changed. (c) SEM images showing the cross-sectional views of the chamber devices prepared using sacrificial NaCl particles with sizes of 15–25 µm.

Fig. 3(a) shows SEM images of the surface of the chambers fabricated using NaCl particles with different size ranges (15–25 and 30–60 µm). Non-spherical pores were formed on the bottom surfaces of these chambers regardless of the chamber size and the sacrificial particle size. The number of the pores on the chambers prepared with smaller sacrificial particles (15–25 µm) was higher than that on chambers fabricated with larger particles (30–60 µm). Because surface pores are generated at the contact points between the mold and faceted sacrificial particles, the use of larger NaCl particles resulted in fewer pores on the chamber bottom surface. In the 100 µm chamber devices fabricated using larger sacrificial particles, pores were not observed on the upper surfaces of the walls separating adjacent chambers. This observation is probably because the larger NaCl particles did not penetrate into the narrow and deep gaps between the convex mold structures corresponding to the chamber walls.

Fig. 3(b) shows the size distributions of the pores formed on the chamber bottoms. In the 50 µm chambers, the average pore diameters were 3.3 ± 2.0 µm (sacrificial particle size: 5–15 µm), 2.9 ± 1.2 µm (15–25 µm), and 7.1 ± 4.0 µm (30–60 µm). A similar trend was observed in the 100 µm chambers, with average pore diameters of 3.7 ± 1.5 µm, 4.3 ± 1.2 µm, and 5.9 ± 3.0 µm, respectively. Despite the substantial increase in sacrificial particle size, the average pore diameter increased by only approximately twofold. This limited increase can be attributed to the fact that the morphology of surface pores is primarily governed by the edges of the non-spherical NaCl particles, rather than by their overall size. Notably, under all fabrication conditions examined, most pores were smaller than typical mammalian cells (10–20 µm in diameter), indicating that the fabricated microchamber devices are suitable for efficient cell trapping while allowing solution permeation. Furthermore, cross-sectional observations revealed that pores were densely formed within the base substrate but were largely absent in the inter-chamber walls (Fig. 3(c)), which is advantageous for selective cell capture within the chambers and controlled vertical flow generation. In our previous study, we confirmed that when the volume fraction of NaCl particles exceeded 45%, nearly complete removal of the introduced NaCl was achieved after the leaching process.^34^ Therefore, in the present device, the pores within the porous substrate are expected to form a fully interconnected network.

### 3.2. Characterization of the porous microchamber device

**Fig. 4:**
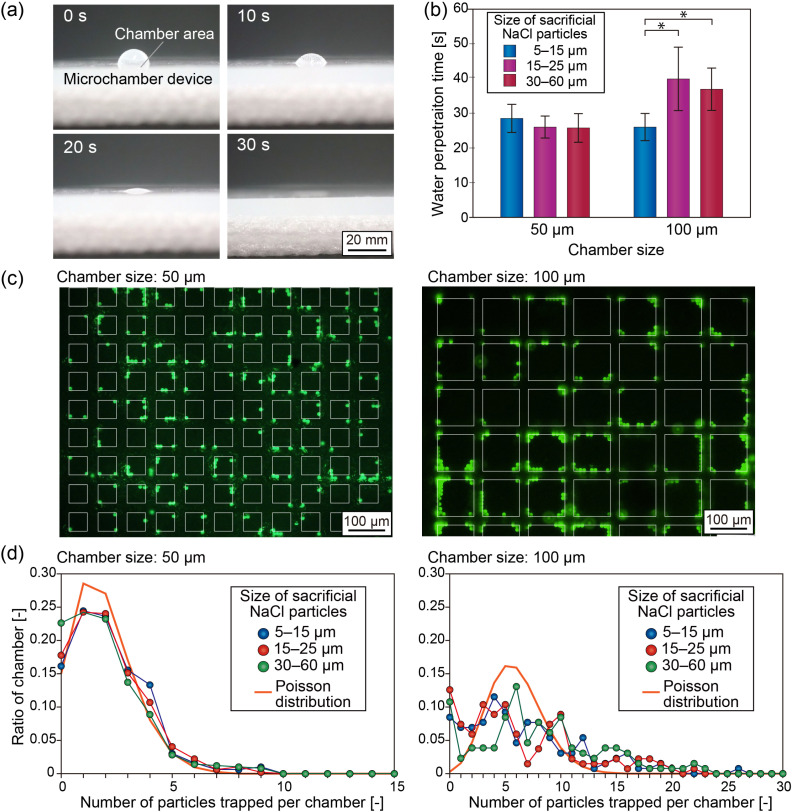
Characterization of the microchamber devices with different size chambers prepared using different-size sacrificial NaCl particles. (a) Water droplet penetrating through the porous substrate of the 50 µm microchamber device, prepared using sacrificial NaCl particles of 15–25 µm. (b) Water permeation tests for the chambers. Data are presented as the mean ± standard deviation from four independent experiments. *p < 0.05 by one-way ANOVA followed by Fisher's LSD test. (c) Capture of 9.9 µm green fluorescent microparticles. (d) Distribution of the numbers of the trapped particles.

We evaluated whether the internal pores of the porous substrate were sufficiently interconnected to enable efficient solution introduction and exchange by simply dispensing liquid samples onto the device. In this experiment, a wipe was placed beneath the device, and the porous substrate was pre-filled with distilled water. A 4 µL droplet of DI water was then dispensed onto the porous area of a device with a chamber size of 50 µm, fabricated using sacrificial NaCl particles with sizes of 15–25 µm. Time-lapse images of the penetration process are shown in Fig. 4(a). The droplet placed on the porous area smoothly infiltrated the porous substrate within approximately 30 s. This smooth solution introduction was driven by capillary force generated by the wipe through the porous substrate. When the wipe was removed, the flow was temporarily stopped. Although PDMS is intrinsically hydrophobic, vertical flow could be reproducibly generated once the porous substrate had been pre-filled with water.

We next examined devices with different chamber sizes fabricated using sacrificial NaCl particles of various sizes by similarly dispensing 4 µL of DI water and measuring the penetration time. This volume was selected as an optimal amount to ensure uniform coverage of the entire chamber area while preventing overflow into the surrounding regions. The results are shown in Fig. 4(b). In all cases, the water droplet permeated the porous substrate within 25–40 s, indicating rapid and reproducible solution introduction. A slight difference in penetration time was observed for the 100 µm chambers prepared using different size NaCl particles. Because the flow resistance of porous PDMS depends on multiple factors, including pore size, density, shape, and connectivity, it is likely that these parameters did not differ substantially among the fabricated devices.

Based on the fluid permeability results, we next evaluated the trapping behavior of model particles with sizes comparable to those of cells (9.9 µm in diameter). In this experiment, 4 µL of DI water containing approximately 1000 fluorescent particles was dispensed onto the porous microchamber array, and the trapped particles were observed using a fluorescence microscope. Representative fluorescence images of devices with chamber sizes of 50 µm and 100 µm fabricated using sacrificial NaCl particles with sizes of 15–25 µm are shown in Fig. 4(c). For both chamber sizes, most of the introduced particles were captured within the microchambers, while only a limited number were observed on the top surfaces of the inter-chamber walls. The ratio of particles trapped inside the chambers relative to the total number of particles observed within the porous area exceeded 90% for both chamber sizes. A small fraction of particles was occasionally observed on the surface of the silicone sheet outside the porous area, likely due to the uneven application of the particle suspension during manual dispensing.

Within the chambers, particles tended to accumulate near the inner walls, and this tendency was more pronounced in the 100 µm chambers. It is probable that, at the final stage of fluid infiltration through the porous substrate, meniscus formation induces particle accumulation near the chamber boundaries. Fig. 4(d) shows the distribution of the number of particles trapped per chamber, as quantified from the fluorescence images. For the 50 µm chambers, similar trapping behavior was observed regardless of the size of the sacrificial NaCl particles, and the number of trapped particles closely followed a Poisson distribution. In contrast, particle trapping in the 100 µm chambers deviated from Poisson statistics. This deviation is likely attributable to both the smaller number of chambers, which increases statistical uncertainty, and the presence of relatively large pores formed in the chamber walls, which may induce non-uniform flow patterns. Based on these results, 50 µm chambers were employed in the following experiments for quantitative determination of cell populations.

### 3.3. Staining of intracellular molecules

Protocols for intracellular molecule staining generally require multiple chemical processing steps. In this study, actin was selected as a representative intracellular target and stained using fluorescence-labeled phalloidin in both adherent and suspension cells. In addition, cell nuclei were simultaneously stained with Hoechst 33342. Cell capture and staining were performed by sequentially dispensing 4 µL aliquots of cell suspension, fixative solution, permeabilization reagent, and dye solution onto the device. Between each step, the device was washed two or three times with a washing buffer to minimize cross-contamination of reagents. During the staining procedure, a wipe was placed beneath the device in contact with the porous substrate to induce vertical flow. Because reagent penetration occurred relatively rapidly, the wipe was removed before complete infiltration of the applied solutions to prevent cell drying. In addition, the device was placed in a Petri dish during each incubation step to suppress evaporation and maintain a humid environment.

**Fig. 5:**
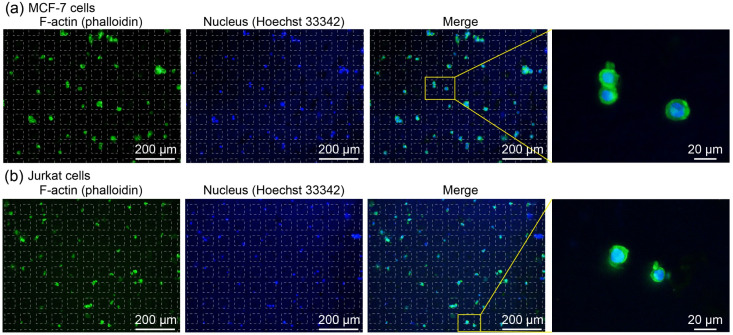
Fluorescence micrographs showing the double-stained cells. F-actin and nuclei were stained with phalloidin (green) and Hoechst33342 (blue), respectively. (a) MCF-7 cells, and (b) Jurkat cells. The positions of the chambers (50 µm) are shown with dashed white lines.

Fig. 5(a) shows representative fluorescence images of adherent MCF-7 cells after staining. In contrast to the case of fluorescent particles, no clear tendency for cells to accumulate near the chamber walls was observed. The green fluorescence signal derived from F-actin and the blue fluorescence signal from the nuclei were spatially co-localized, indicating successful dual staining. Magnified images further revealed that the cells retained their circular morphology, indicating that no obvious morphological changes were observed after the capture and staining processes. In general, the permeabilization process for cell membranes using surfactants can compromise membrane integrity and cellular stability. In the present system, however, the elastic nature of the porous PDMS microchambers likely reduced mechanical stress for cells during processing, contributing to the preservation of cell morphology. The staining results for suspension Jurkat cells are shown in Fig. 5(b). Similar to the adherent cells, clear dual staining of F-actin and nuclei was achieved. For both cell types, circularity, projected area, and mean fluorescence intensity were quantified and compared with those obtained using the conventional centrifugation-based solution exchange method. The results are presented in Fig. S1. No significant differences were observed between the proposed device and the conventional method for these parameters, demonstrating that the proposed microchamber device is applicable to both adherent and suspension cell types. These results indicate that the device enables intracellular staining across a broad range of cell types with distinct characteristics.

The cell capture efficiencies were 91.7 ± 7.0% for MCF-7 cells and 90.1 ± 7.2% for Jurkat cells, indicating minimal cell loss during the staining process and showing the potential of the system for rare cell analysis. Conventionally, staining of adherent cells is performed on cells attached to culture substrates, whereas staining of suspension cells typically relies on centrifugation-based solution exchange. In the latter case, substantial cell loss can occur due to repeated centrifugation and manual pipetting, with reported recovery efficiencies of approximately 60%.^18^ In the present study, on the other hand, staining could be performed simply by dropping a small amount of staining reagents and washing buffer onto the device, indicating that the system not only reduces reagent consumption and cost but also allows convenient and efficient staining without the need for additional complex operations. In addition, the proposed method is more compatible with automated systems compared to centrifugation-based procedures. Furthermore, the concept of performing chemical treatment on cells simply by adding a droplet of solution can be extended beyond staining to other biological processes that require multistep sample introductions, such as drug resistance evaluation or immunoresponse assays.

### 3.4. Identification and quantification of cancer cells

Detection of circulating tumor cells (CTCs) from patient blood samples and characterization of their populations have become key research topics in cancer diagnosis and prognosis prediction.^36^ CTCs exhibit pronounced phenotypic heterogeneity,^37^ particularly in the context of epithelial–mesenchymal transition. Because EMT is closely associated with cancer progression and malignancy, it can strongly influence therapeutic strategies. Representative intracellular markers for epithelial and mesenchymal phenotypes include cytokeratin and vimentin, respectively.^38^ When detecting and characterizing CTCs in blood samples, efficient solution exchange operations for cells in suspension are indispensable. Therefore, a microchamber-based device that enables simple and efficient solution exchange is expected to be effective for such applications. In this experiment, Jurkat cells were used as a model for leukocytes, while MCF-7 cells were used as a model for epithelial cell-type CTCs. Staining of the epithelial marker cytokeratin-19 (CK-19) was performed while varying the mixing ratio of these two cell types. Specifically, samples containing 0, 10, or 100 MCF-7 cells mixed with 1000 Jurkat cells were introduced into the microchamber device, and quantitative evaluation of CK-19-expressing cells was conducted. A negative control experiment (no primary antibody) was performed using MCF-7 cells alone, confirming that CK-19 staining was absent in the absence of the primary antibody (Fig. S2).

**Fig. 6:**
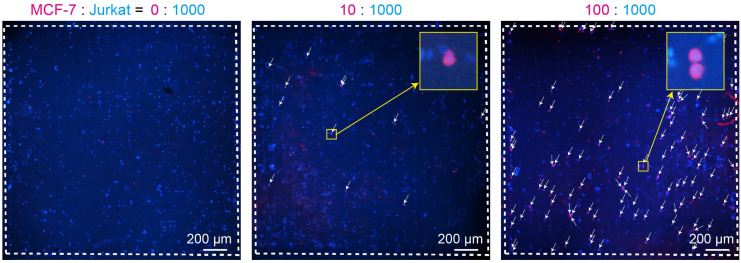
Fluorescence micrographs showing the stained cell mixture of MCF-7 and Jurkat cells. Cytokeratin 19 (CK-19) and cell nuclei were stained with anti CK-19 antibody (red) and Hoechst33342 (blue), respectively. The number of the applied MCF-7 cells was changed, whereas that of Jurkat cells was constantly at 1000 cells. The entire porous areas are shown. Dashed white lines indicate the boundary of the porous area. Identified MCF-7 cells are indicated with white arrows.

Fig. 6 shows the results of dual staining for CK-19 and cell nuclei in heterotypic cell mixtures. Under all conditions, blue fluorescence corresponding to cell nuclei was observed throughout the porous area containing 529 chambers. Although the cells were not distributed completely uniformly, this nonuniformity is likely attributable to variations in manual sample dispensing, as well as to inherent heterogeneity in pore distribution within the device. When MCF-7 cells were added, red fluorescence originating from CK-19 was clearly observed in conjunction with nuclear staining, demonstrating that the microchamber device enables identification of specific cancer cell types. As the number of spiked MCF-7 cells increased, the number of CK-19-positive cells increased accordingly. Magnified images further confirmed that cell morphology was well preserved, consistent with the results described in the previous section. A small number of red fluorescent spots were also detected in samples without introduced MCF-7 cells; however, these signals did not colocalize with nuclear fluorescence. These signals were attributable to nonspecific adsorption of antibodies onto cell debris or other particle-like objects rather than to intact cells.

The number of detected MCF-7 cells was 16.8 ± 4.1 when ten cells were theoretically introduced and 117.4 ± 9.3 when one hundred cells were introduced, indicating that the system enables reasonably accurate quantitative evaluation of rare cell populations. Although only two cell lines were examined in this study, the same strategy could be extended to the detection of multiple cell types. For example, epithelial and mesenchymal populations could be distinguished by simultaneously employing multiple staining probes, such as phenotype-specific antibodies. Furthermore, while the chamber array in this study was confined to a 2 mm × 2 mm porous region for ease of observation, the proposed approach is readily scalable. The number of chambers can be tuned according to the sample volume and the expected proportion of target cells, further enhancing the applicability of the system to rare cell analysis.

## 4. Conclusion

In this study, we developed a multistep cell-processing device incorporating an array of porous microchambers capable of generating vertical flows, and demonstrated a simple and efficient strategy for cell capture and intracellular molecule staining. By employing an unconventional fabrication process that combines replica molding with salt-leaching techniques, porous microchamber devices with micropores comparable to or smaller than the size of mammalian cells were reproducibly fabricated without the need for complex microfabrication procedures. By controlling the size of sacrificial NaCl particles and the chamber dimensions, conditions suitable for effective cell trapping were investigated. Using the optimized device, we successfully achieved specific staining and visualization of intracellular molecules in both adherent and suspension cells, as well as quantitative evaluation of rare cell populations. Simple drop-based application of cell suspensions, cell processing reagents, and washing buffers enabled highly efficient solution exchange, completely eliminating the multiple rounds of centrifugation required in conventional protocols for intracellular molecule staining. As a result, cell loss was minimized while operational complexity and reagent consumption were substantially reduced.

In the present device, the number of pores formed in each chamber was limited for cell capture. Future studies will focus on identifying fabrication conditions that enable the formation of a greater number of smaller pores, as well as on optimizing chamber geometries and dimensions to further improve cell trapping efficiency and handling performance. In addition, although pre-wetting of the internal pores of the hydrophobic PDMS substrate was required in this study, operability could be further enhanced by employing hydrophilization strategies of the porous substrate, for example, through the incorporation of hydrophilic polymers.^39,40^

Furthermore, the porous substrate is optically opaque, which makes bright-field observation challenging. Therefore, future device designs may benefit from reducing the substrate thickness to improve optical transparency. On the other hand, if pore size can be more precisely controlled, the platform may enable direct isolation of cancer cells from complex biological samples such as whole blood.^41^ In addition, incorporation of strategies for cell retrieval would allow integration with downstream analytical processes, such as flow cytometry. Through these refinements, the proposed microchamber-based device is expected to be widely adopted as a facile and versatile platform for intracellular molecule visualization and multistep cell processing in biological research.

## Author contributions

A. O.: investigation, formal analysis, writing – original draft, writing – review & editing. Y. A.: conceptualization, investigation, methodology, formal analysis, writing – review & editing. R. U.: investigation, writing – review & editing. M. Y.: conceptualization, investigation, methodology, writing – original draft, writing – review & editing. All authors have read and agreed to the published version of the manuscript.

## Conflicts of interest

There are no conflicts to declare.

## Supplementary Material

RA-016-D5RA10009G-s001

## Data Availability

Raw data supporting the findings of this study are available from the corresponding author upon reasonable request. Supplementary information (SI): Fig. S1 and S2. See DOI: https://doi.org/10.1039/d5ra10009g.
