# Supplementary material for: Vertical-flow porous microchamber arrays for cell capture, intracellular molecule staining, and population analysis
Source: RSC Adv. 2026 Apr 22;16(23):20923–32. doi: 10.1039/d5ra10009g (PMC13101962; doi:10.1039/d5ra10009g)
Supplement: RA-016-D5RA10009G-s001 [file RA-016-D5RA10009G-s001.pdf]

Supplementary Information (SI) for RSC Advances.
This journal is © The Royal Society of Chemistry 2026

Electronic supplementary information

Vertical-flow porous microchamber arrays for cell capture, intracellular molecule staining, and population analysis

*Asahi Ohtsu†, Yusuke Araki†, Rie Utoh and Masumi Yamada**

Department of Applied Chemistry and Biotechnology, Graduate School of Engineering, Chiba University, 1-33 Yayoi-cho, Inage-ku, Chiba 263-8522, Japan.

*E-mail: m-yamada@faculty.chiba-u.jp; Tel & Fax: +81-43-290-3398

†These authors contributed equally to this work.

List of Supporting Information

Figure S1. Comparison of cell staining performance between the conventional method and the proposed microchamber device.

Figure S2. CK-19 staining of MCF-7 cells on the microchamber device.

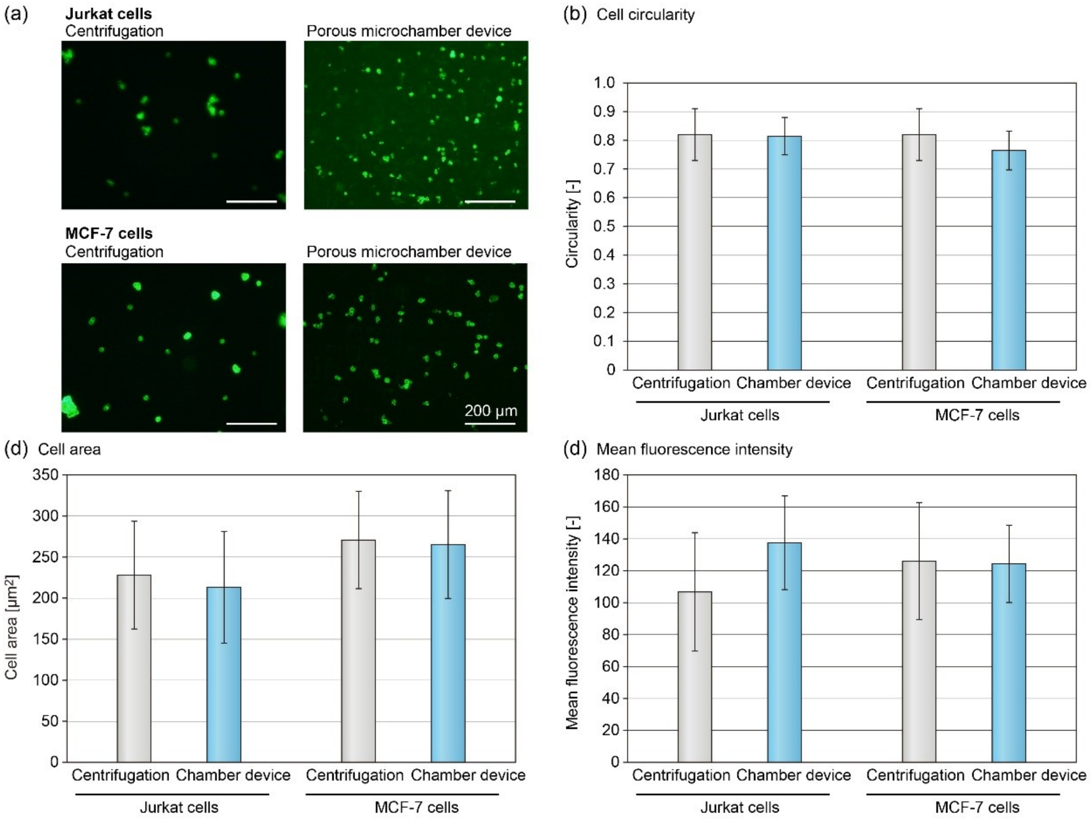

Figure S1. Comparison of cell staining performance between the conventional method and the proposed microchamber device. (a) Representative fluorescence micrographs of Jurkat and MCF-7 cells with F-actin stained using fluorescence-labeled phalloidin. Cells were processed either by the conventional centrifugation-based solution exchange method or by the proposed porous microchamber device. (b) Circularity, (c) projected cell area, and (d) mean fluorescence intensity under the indicated conditions. In panels (b)–(d), an average of 75 cells were analyzed for each condition.

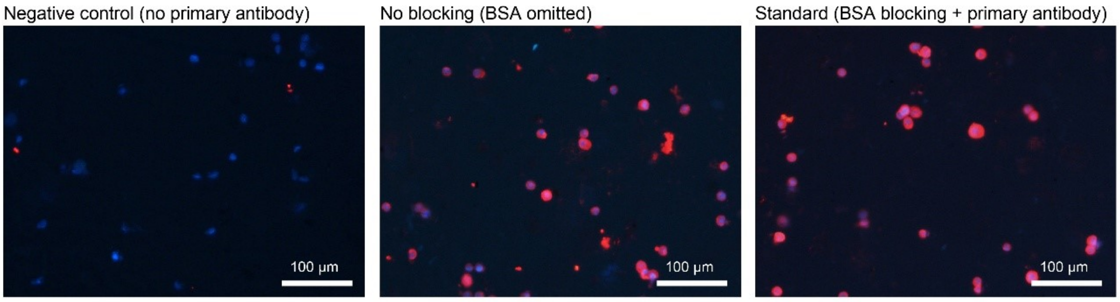

Figure S2. CK-19 staining of MCF-7 cells on the microchamber device. (Left) No primary antibody control; (middle) staining performed without BSA blocking; (right) standard staining condition.
